# 91 The BoBS Score - A Novel Machine Learning Based Score for Predicting Burn Survival

**DOI:** 10.1093/jbcr/iraf019.091

**Published:** 2025-04-01

**Authors:** Sonja Schmidt, Marius Drysch, Jannik Hinzmann, Flemming Puscz, Felix Reinkemeier, Marcus Lehnhardt, Christoph Wallner

**Affiliations:** BG Universitaetsklinikum Bergmannsheil Bochum; BG Universitaetsklinikum Bergmannsheil Bochum; BG Universitaetsklinikum Bergmannsheil Bochum; BG Universitaetsklinikum Bergmannsheil Bochum; BG Universitaetsklinikum Bergmannsheil Bochum; BG Universitaetsklinikum Bergmannsheil Bochum; BG Universitaetsklinikum Bergmannsheil Bochum

## Abstract

**Introduction:**

Predicting burn mortality remains a critical aspect of burn care, guiding clinical decision making. Recent studies have highlighted the limitations of traditional scores such as Baux or ABSI, and modified versions of existing scores have been introduced over time. Advances in burn care - including wound care, resuscitation protocols and critical care practices - underscore the need for updated prediction models that reflect modern treatment. In particular, the potential of new technologies, particularly machine learning, offers the opportunity to analyse large real-world data sets and thus to reflect clinical developments in burn care. This research aims to develop an innovative mortality prediction score that uses artificial intelligence to incorporate new advancements in medicine.

**Methods:**

In this study, a variety of machine learning methods were used to analyse big data from a national burn registry (over 10,000 patients) regarding the primary endpoint of mortality. XGBoost was used to identify the most relevant features and the scoring model was developed using the top 10 features identified by the XGBoost model. The weights for each feature were calculated based on the importance of the feature and the values of the corresponding variable. The importance scores were rescaled to a 0-10 scale to create a more interpretable and clinically relevant scoring system. A weighted score was assigned for binary features, and a scoring system based on calculated intervals was implemented for continuous variables. The final model was evaluated using 5-fold cross-validation to minimise the log loss metric.

**Results:**

The ten most relevant factors for mortality were identified as TBSA, full thickness burns, risk factor ‘pre-existing kidney disease’, age, inhalation injury, risk factor ‘diabetes’, ‘COPD’, cooling, superficial partial thickness burns and ‘heart failure’. These factors have been incorporated into a 0-1 point scoring system which is highly effective with an accuracy of 93.1% and a ROC AUC of 92.4%. Relevant dependencies between TBSA and age and between TBSA and full-thickness burns were also described.

**Conclusions:**

This score represents a modern, machine-learning-based approach to predicting burn mortality that offers improved accuracy over traditional scores, yet is easy and straightforward to use in clinical practice. Despite some limitations, the score holds great promise for guiding clinical decisions and optimising burn care. This score is the first machine learning based mortality score in burn care, and the first to include multiple risk factors, but it still needs further validation and advancements in the future, however, it is a sufficient tool to work with.

**Applicability of Research to Practice:**

This study presents a burn survival prediction score that is easy to use in clinical practice and can therefore help guide clinical decision making.

**Funding for the Study:**

N/A

